# From infection to dysfunction: viral triggers and antiviral immune factors in Alzheimer’s disease pathology

**DOI:** 10.3389/fimmu.2026.1839357

**Published:** 2026-06-19

**Authors:** Soo-Jin Oh, Ok Sarah Shin, Ji-Yeun Hur

**Affiliations:** 1 Brain Korea 21 (BK21) Graduate Program, Department of Biomedical Sciences, College of Medicine, Korea University Guro Hospital, Seoul, Republic of Korea; 2 Vaccine Innovation Center, College of Medicine, Korea University, Seoul, Republic of Korea; 3 Department of Biological Sciences, Ajou University, Suwon, Republic of Korea

**Keywords:** Alzheimer’s disease, antiviral factors, neurodegeneration, neurotropic viruses, viral infection

## Abstract

Neurodegenerative diseases and neurocognitive disorders increasingly appear to share a common and underappreciated contributor: the viral-immune axis in the brain. This review presents current evidence linking neurotropic viruses and host antiviral immunity to the onset and progression of neurodegeneration and neurocognitive dysfunction. We explore how viral infections, particularly by Herpesviruses, Severe Acute Respiratory Syndrome Coronavirus 2, and Human Immunodeficiency Virus, disrupt neural homeostasis through neuroinflammation, amyloidosis, tauopathy, and autophagy dysregulation in neurodegeneration including Alzheimer's disease (AD). Simultaneously, host antiviral mechanisms, including type I interferons and interferon regulatory factors, often amplify neuronal damage when dysregulated. By examining viral and immune interactions within the neurodegenerative diseases, this review aims to broaden our understanding of the viral-immune axis in the brain and inspire novel approaches to prevention and treatment.

## 1 Introduction

### 1.1 Neurotropic viruses-immune axis in the brain

Neurotropic viruses, such as herpes simplex virus type 1 (HSV-1), varicella zoster virus (VZV), severe acute respiratory syndrome coronavirus 2 (SARS-CoV-2), and human immunodeficiency virus type 1 (HIV-1) may contribute to the risk and progression of neurodegenerative diseases. Neurotropic viruses are likely to infiltrate the central nervous system (CNS) via multiple routes, including the olfactory nerve and a “Trojan Horse” mechanism involving infected immune cells (**Table 1**) (1). Vascular pathways such as endothelial cell infection and disruption of the blood-brain barrier (BBB) and blood-cerebrospinal fluid barrier further enable viral entry into the CNS, often progressing via retrograde axonal transport. Viral infections of the brain can cause inflammatory conditions such as meningitis, encephalitis, and meningoencephalitis. Herpes simplex virus encephalitis (HSE), commonly triggered by HSV-1 reactivation, exemplifies how viral-induced neuronal cell death and release of damage-associated molecular patterns (DAMPs) can provoke widespread neuroinflammatory cascades in the brain (2). This periodic reactivation from latency acts as a chronic stimulus, perpetuating low-grade neuroinflammation and progressively driving the accumulation of neurodegenerative hallmarks.

**Table 1:** Characterization of viral neuroinvasion and neuropathogenesis in the central nervous system.

Virus	CNS complications	Target cells of the brain	Neuroinvasion mechanisms	Molecular mechanisms for neuropathogenesis
**HSV-1**	AD, HSE	Neurons, Astrocyte, Microglia	Retrograde axonal transport	Induction of neuronal apoptosis, necrosis, and cytolysis
				Activation of glia-mediated neuroinflammation
				Promotion of Aβ aggregation and tau hyperphosphorylation
				Dysregulation of autophagic and mitophagic flux
**VZV**	Encephalitis, Meningitis, Cerebellitis, Myelitis, Dementia	Neurons, Endothelial cells, Oligodendrocyte, Microglia	Retrograde axonal transport	Induction of demyelination
				Activation of glia-mediated neuroinflammation
				Establishment of latent infection in the dorsal root ganglion
**SARS-CoV-2**	Cognitive impairments, AD, Encephalopathy	Endothelial cells, Choroid plexus epithelial cells, Microglia, Pericytes	Olfactory nerve route, BBB disruption	Activation of glia-mediated neuroinflammation
				Hypoxia-induced neuronal injury
				Microthrombosis-driven neurodegeneration
				Promotion of Aβ aggregation and tau hyperphosphorylation
				Systemic cytokine storm
**HIV**	HAND, ANI, MND, HAD	Microglia, Perivascular macrophages, Astrocyte	Trojan horse mechanisms	Indirect induction of neuronal apoptosis
				Promotion of Aβ aggregation and tau hyperphosphorylation
				HIV protein-mediated (Tat, gp120) excitotoxicity

CNS, central nervous system; HSV-1, herpes simplex virus type 1; AD, Alzheimer's disease; HSE, herpes simplex encephalitis; Aβ, amyloid β; VZV, varicella zoster virus; SARS-CoV-2, severe acute respiratory syndrome coronavirus 2; BBB, blood-brain barrier; HIV-1, human immunodeficiency virus type 1; HAND, HIV-associated neurocognitive disorders; ANI, asymptomatic neurocognitive impairment; MND, mild neurocognitive disorder; HAD, HIV-associated dementia.

Uncontrolled immune responses within the CNS, which is marked by immune cell infiltration and activation of resident glia, lead to severe tissue damage and amplify pathogenesis. Beyond acute inflammation, associations between neurotropic viruses and neurodegenerative outcomes are increasingly documented, with HSV-1 implicated in Alzheimer’s disease (AD) (3–5). Elucidating how CNS homeostasis is compromised by persistent viral infections is essential to understanding disease development and designing targeted interventions. In this review, we introduce how neurotropic viruses may serve as potent extrinsic modulators of neurodegeneration and summarize mechanisms of neurotropic viral contribution to the pathogenesis of neurodegenerative diseases.

### 1.2 Antiviral immunity in the CNS

When neurotropic viruses infiltrate anatomical barriers in the brain, the innate immunity kicks in upon the entry of pathogens as a host defense mechanism (6). Regardless of different entry routes of DNA or RNA viruses, neurotrophic viruses eventually reach the brain parenchyma which is mainly made of neuronal, astrocyte, and microglial cell populations (7–9). Neurons are specialized cells that transfer information in synaptic transmission and are characterized as a non-renewable cell population (9). Therefore, neurons are vulnerable to insult, and especially aged neurons are more vulnerable to damage (6). Astrocytes and microglia are collectively referred to as glia or glial cells. Microglia cells have myeloid origin like macrophages and are brain resident sentinel cells surveillancing the environment, removing unwanted cell debris or foreign pathogens by phagocytosis, and carrying out synaptic pruning during brain development. Astrocytes are also immune-like cells in the brain, nourish neurons by producing neural growth factors, and keep a homeostatic environment around neurons by spatial buffering such as regulating external potassium concentration (9, 10). Neurons, astrocytes, and microglia all utilize type I interferon (IFN) production as a major defense mechanism against viral infection (9). Since some neurons have lower levels of IFN, glial cells carry most antiviral responses to infection, and neurons also apply different antiviral strategies such as viral clearance by autophagy (6, 9). Additionally, antiviral innate immunity utilizes epigenetic regulation, including chromatin remodeling (e.g., DNA methylation), histone modification, and RNA modification during host-virus interactions (11). Chromatin targeting restriction factors, such as PML-NBs, KRAB/KAP1, IFI16, and HUSH complex, together with other epigenetic modifiers, recruit histone marks (e.g., H3K9me3) on viral genomes, resulting in more compact viral chromatin and decreased viral transcription (12–14). Therefore, repressing viral DNA prevents lytic viral replication and cell death in host cells, such as neurons. However, this also allows viruses to establish latent infection in neurons, as shown in HSV-1 (15). Neuron-specific microRNAs (miRNAs) such as miR-138 and miR-9 decrease viral gene expression and the replication of HSV-1 in cell culture, as viral epigenetic silencing, which promotes viral latency (16, 17).

**Figure 1:**
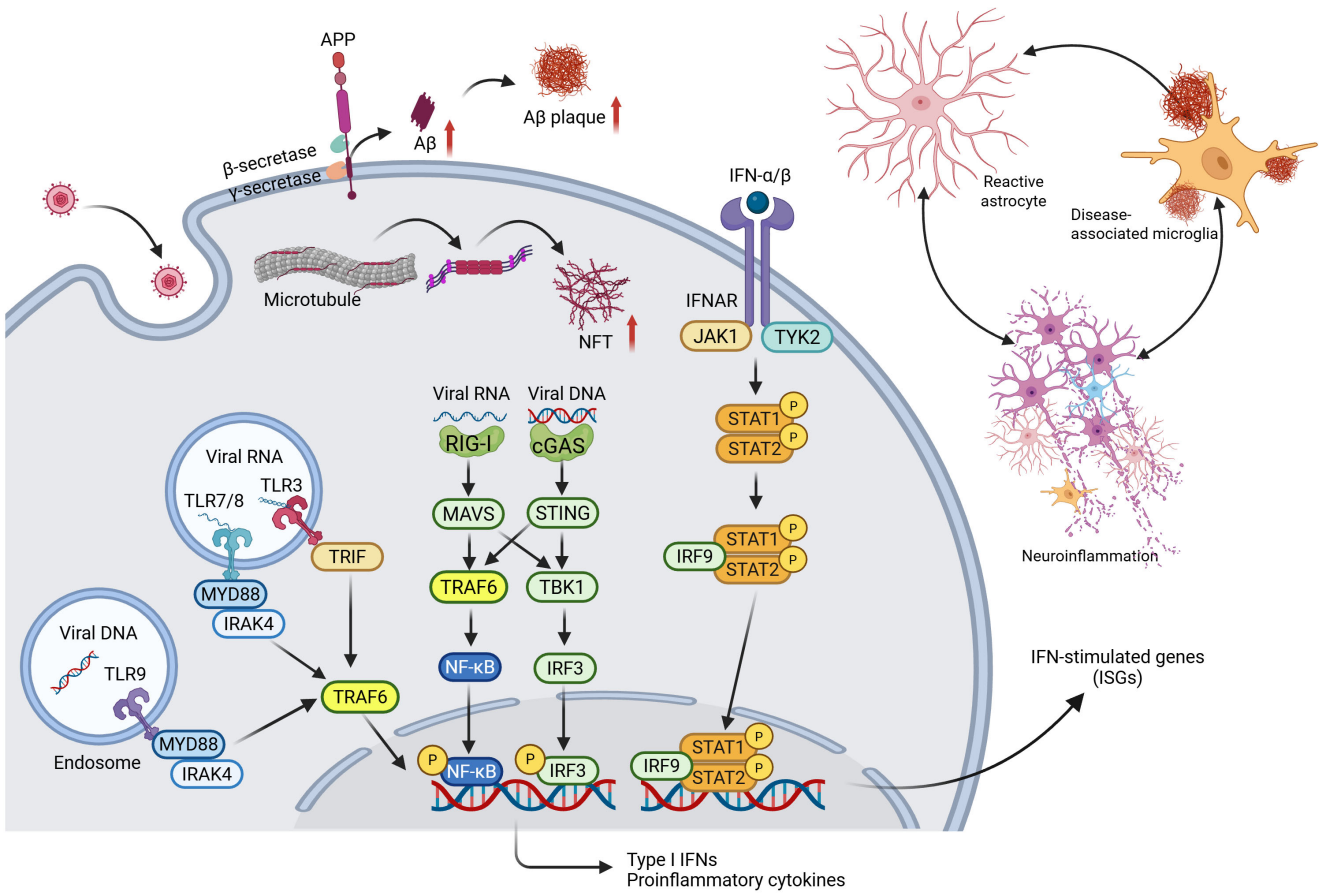
Antiviral immunity in the central nervous system. Intracellular viral components are detected by endosomal (TLR3/7/8/9) and cytosolic (cGAS-STING and RIG-I-MAVS) pattern recognition receptors. Activation of these pathways triggers downstream signaling cascades, leading to the nuclear translocation of IRF3 and NF-κB, which induce the production of type I interferons (IFN) and proinflammatory cytokines. Concurrently, extracellular IFN signaling through the IFNAR-JAK1-TYK2 pathway activates the ISGF3 (STAT1/STAT2/IRF9) complex, resulting in the robust upregulation of IFN-stimulated genes (ISGs). Ultimately, reactive astrocytes, disease-associated microglia, and the sustained release of proinflammatory cytokines drive chronic neuroinflammation and exacerbate neurodegeneration.

As a primary component of the innate immune response, the signatures of viruses, including viral structures or nucleic acids, are recognized by pattern recognition receptors (PRRs) on the surface or in the cytosol of infected host cells (9). These PRRs recognize pathogen-associated molecular patterns (PAMPs) or microbe-associated molecular patterns (MAMPs), which in turn activate signaling cascades to trigger antiviral IFN responses (9, 18). For instance, the Toll gene was identified in *Drosophila*. The mammalian equivalent of Toll-like receptors (TLRs) is one of the PRRs, which are localized at the plasma membrane or in endosomes (9, 18, 19). Mammalian PRRs activate transcriptional factors (NF-κB, interferon regulatory factor; IRF), phosphorylated transcriptional factors are translocated into the nucleus, and type I IFNs and proinflammatory cytokine (interleukin; IL) genes are expressed (18). Another mammalian PRR, RIG-I-like receptors (RLRs), are the cytosolic sensor for viral RNA. The interaction of the mitochondrial adaptor protein MAVS with RIG-I triggers signaling cascades by activating TBK1. TBK1 phosphorylates transcriptional factors (NF-κB, IRF), which in turn induce antiviral gene expression for proinflammatory cytokines (**Figure 1**) (18). When a DNA virus is endocytosed, viral DNA in the cytosol is detected by cGAS-STING pathway. cGAS is a sensor for cytosolic DNA in the cells and signals via STING, an ER membrane adaptor. cGAS-STING complex migrates to the Golgi, phosphorylates a transcription factor IRF3, in turn induce type I IFNs (9, 20). However, neurotropic viruses have also evolved several strategies to bypass these host innate immune systems by limiting each step. If the host fails to clear these pathogens during the acute phase, they may establish a persistent or latent infection (8).

IFNs produced by infected host cells can bind to IFN receptors in own or nearby cells, which in turn initiate IFN signaling pathways to induce IFN-stimulated genes (ISGs). The binding of IFNs (type I IFN (IFN-α, IFN-β), type II (IFN-γ), and type III (IFN-λ)) to their IFN receptors causes phosphorylation of Janus kinases (JAK1, JAK2, and tyrosine kinase 2 (TYK2)) at the cytoplasmic domains of IFN receptors, which in turn activate STAT proteins (STAT1, STAT2). These STAT complexes translocate into the nucleus, and synthesized ISGs (8). The expression of ISGs serves as a potent cellular defense, with over 1, 000 distinct ISG proteins identified to date. For example, tetherin at the plasma membrane interferes with the tethering of HIV (18). Interferon-induced protein 3 (IFITM3) blocks fusion during viral entry between cell membranes and endosomes (21). In addition, IFITM3 was reported to bind to and modulate γ-secretase, leading to an increase in amyloid β (Aβ) production in aging animal models, AD transgenic models, and AD human brains, suggesting a role for neuroinflammation in AD (22).

## 2 Neurotropic viruses in neurodegenerative pathology

### 2.1 Herpesviruses

HSV-1 is known to infect around 80% of people by the age of 60 and establishes lifelong latency in the trigeminal ganglion with episodic reactivation (2). Since 1980s, accumulating evidence has suggested a close link of HSV-1 with the etiology of neurodegenerative disease, especially AD (3–5). For example, HSV-1 DNA has been detected in amyloid plaques, and sequence homology between HSV-1 glycoprotein B and Aβ has led to the hypothesis that HSV-1 may contribute to plaque nucleation or aggregation (23). This hypothesis is further supported by findings from cellular and animal models showing that HSV-1 infection can enhance Aβ accumulation and tau hyperphosphorylation, although these preclinical systems do not fully recapitulate the complexity of human AD pathology (24–26). Epidemiologically, HSV-1 infection is associated with a 2- to 3-fold increase in AD risk among individuals carrying the apolipoprotein E ϵ4 (APOE4) allele, whereas APOE4-negative individuals show no significant correlation (adjusted hazard ratio (HR) ~0.92) (27). Interestingly, recent neuroimaging studies revealed that HSV-1-infected individuals exhibit distinct microstructural changes in the parahippocampal cingulum and fornix, regions critical for memory processing and cognitive function (28). Elevated anti-HSV-1 immunoglobulin G (IgG) levels have been associated with structural brain abnormalities, including reduced hippocampal volume, particularly in APOE4 carriers. However, these associations should be interpreted cautiously, as anti-HSV-1 IgG represents an indirect marker of prior exposure or systemic immune response and may not reliably reflect active viral reactivation within the CNS (28).

Meanwhile, a recent study reported that individuals with a history of HSV-1 diagnosis were associated with an approximately 1.8-fold increased risk of subsequent AD diagnosis (29). In particular, this risk escalates to approximately 2.1-fold in those aged over 75, suggesting that age may be an important modifying factor in the association between HSV-1 infection and neurodegenerative risk. Interestingly, individuals receiving anti-herpetic treatment were associated with a reduced risk of AD (adjusted HR ~0.80), highlighting the potential for neuroprotective effect of antivirals targeting HSV-1. In addition, about 50-75% of viral cases causing viral encephalitis are due to HSV, and resulting HSE affects children as well as older persons (30). HSE is treated with intravenous acyclovir every 8 hours at a dose of 10 mg/kg for 14 days or 21 days in immunocompromised patients (30, 31). Although fatality rate decreases with antiviral therapy, surviving patients display severe cognitive and psychiatric sequelae such as cognitive impairments, memory loss (anterograde/retrograde amnesia), seizures, delusions, hallucinations, etc. (32) The magnetic resonance imaging (MRI) shows brain abnormalities in the medial temporal lobes (e.g., hippocampus), the insular, cingulate, and frontobasal cortex (31, 32).

Similar to HSV-1, VZV causes lifelong latency in dorsal root ganglia. Importantly, VZV reactivation can result in herpes zoster and postherpetic neuralgia, a chronic neuropathic pain syndrome that underscores the virus’s capacity to persist and cause neurological dysfunction. A recent meta-analysis reported that VZV infection has been found to be associated with an average 11% increased risk of developing dementia, while antiviral therapy was shown to reduce this risk by approximately 15%, suggesting a possible preventative role for pharmacologic intervention (33, 34). Interestingly, individuals co-infected with both VZV and HSV-1 exhibited a dramatically higher risk of AD, approximately 50%, compared to individuals infected with either virus alone (VZV: 38%, HSV-1: 41%). This finding raises the possibility of a synergistic interaction, although the underlying mechanisms and the contribution of viral latency or reactivation remain to be established.

Recent studies have uncovered a compelling association between vaccination against VZV and a reduced risk of AD especially in older populations (35). Notably, administration of the live-attenuated *Zostavax* vaccine led to a 3.5% reduction in dementia diagnosis within 7 years, with a particularly a stronger protective effect observed in females (35). Complementing this, Taquet et al. reported that a recombinant VZV vaccine, *Shingrix*, provided protection against dementia within six years of vaccination, demonstrating enhanced efficacy in female recipients compared to males (36). Taken together, these observations suggest a possible association between VZV vaccination and reduced dementia risk, although causal mechanisms remain unclear.

Another herpesvirus, cytomegalovirus (CMV), is increasingly recognized as a significant contributor to the inflammatory milieu of the aging brain. Similar to other herpesviruses, CMV establishes a lifelong latent infection, characterized by periodic subclinical reactivations associated with immunosenescence. Chronic CMV infection is associated with inflammaging, a state of chronic, low-grade systemic inflammation. This process is driven by the massive expansion of CMV-specific CD8^+^ T cells, which can account for up to 10–20% of the total T-cell pool in older adults. These cells release proinflammatory cytokines, such as IFN-γ and TNF-α, which can compromise the integrity of BBB and exacerbate neuroinflammatory responses. CMV-induced inflammation may promote the aggregation of Aβ and tau phosphorylation by activating microglia into a proinflammatory phenotype. In neonatal infection models, CMV drives long-term dysfunctional disease-associated microglia (DAM), which are also found in AD brains. These DAM persist into adulthood and contribute to neurodegeneration even in the absence of active viral replication (37, 38).

### 2.2 SARS-CoV-2

Coronavirus Disease 2019 (COVID-19), caused by SARS-CoV-2, has revealed profound neurological implications beyond its respiratory manifestations. Longitudinal studies conducted in China report that over 25% of COVID-19 survivors experience cognitive impairments collectively referred to as “brain fog,” encompassing deficits in attention, memory, and speech (39, 40). These symptoms, commonly observed in long COVID patients, reflect a set of mild to moderate neurological symptoms (41, 42). Individuals diagnosed with COVID-19 face a substantially elevated risk of developing AD, particularly among elderly females over 85 years who suffered severe infections (43, 44). Specifically, while the incidence of AD diagnosis in uninfected individuals is approximately 0.35%, it rises to 0.68% among those previously infected with COVID-19. This substantial increase in AD risk, even with relatively low baseline incidence, raises concern that COVID-19 may contribute to increased neurodegenerative vulnerability in susceptible populations. Elderly females showed a HR of 1.8, and older individuals (>65 years) exhibited a higher incidence of AD overall. COVID-19 vaccination has been shown to significantly reduce mortality and serious cardiovascular complications for at least one year following SARS-CoV-2 infection (45). A recent human clinical study based on blood proteomic analysis showed that individuals with cognitive impairment following mild COVID-19 exhibited significant downregulation of key proteins involved in synaptic regulation and neurotransmission, including WASF3, ARHGAP1, ADD1, EIF4B, and SH3GLB2. This molecular dysregulation was directly associated with structural neurodegenerative changes observed on brain MRI, particularly cortical thinning in regions such as the cingulate and insular cortices, reflecting impaired synaptic maintenance, reduced neuronal connectivity, and diminished functional brain reserve (46). Thus, the pathophysiology of long COVID and sustained neuroinflammation should be further investigated as a risk factor for AD.

### 2.3 HIV

HIV is a retrovirus that primarily infects microglia and other CNS-resident or infiltrating immune cells rather than neurons directly, triggering inflammatory and neurotoxic cascades that may contribute to neurodegeneration. These neurological complications are collectively referred to as HIV-associated neurocognitive disorders (HAND), which encompass a spectrum ranging from asymptomatic neurocognitive impairment (ANI) and mild neurocognitive disorder (MND) to HIV-associated dementia (HAD) (47, 48). Prior to the widespread availability of antiretroviral therapy (ART), the risk of developing HAD was strongly correlated with advancing HIV disease, with incidence rates reaching ~30% in untreated individuals (49).

Studies aiming to identify major risk factors for HAD have indicated that a low CD4^+^ T cell count, older age, underlying comorbidities such as cardiovascular disease or hepatitis C virus co-infection, and suboptimal ART efficacy are key predictors of HAD (50). Importantly, a CD4^+^ T cell count below 200 cells/μL is strongly associated with increased risk of HAD, reflecting the effect of persistent HIV infection on the systemic and CNS immunosuppression (51). Although the introduction of ART has reduced the incidence of HAD to below 10%, ART-associated neurotoxicity including peripheral neuropathies and cognitive side effects have been reported, highlighting the delicate balance between viral suppression and long-term neurological outcomes (52). Thus, continued investigation is essential to unravel the mechanisms underlying HIV-1-induced neuropathogenesis and develop potentially effective therapeutic strategies and manage long-term neurological complications in HIV-infected individuals.

## 3 Possible mechanisms of viral triggers in neurodegeneration

### 3.1 Neuroinflammation

Glial cells are the primary gatekeepers of the CNS during viral infections, playing crucial roles in producing cytokines/chemokines and clearing extracellular pathogens through phagocytosis. In their resting state, astrocytes and microglia actively surveil the CNS to maintain homeostasis and rapidly respond to neural injuries (53). Notably, disease-associated glial phenotypes, such as disease-associated astrocytes (DAAs) and DAM, have been identified in various neurodegenerative conditions, including AD. Given that dysregulation of glial phenotypes correlates with functional impairment and accelerates neurodegeneration, it is important to elucidate how neurotropic viruses trigger glial phenotype shifts, particularly in astrocytes and microglia.

HSV-1, in particular, has been reported to activate microglia and promote a transition toward DAM-like phenotypes in experimental models. HSV-1 infection leads to increased microglial soma area and volume, along with a reduction in process length and branching complexity (54). Importantly, HSV-1-infected microglia also exhibit phagocytic dysfunction and has been associated with impaired Aβ clearance in experimental systems (55). Furthermore, experimental studies utilizing a range of systems, from primary cell cultures and organotypic brain slices to *in vivo* murine models of ocular HSV-1 infection, have demonstrated that the virus activates the cGAS-STING pathway to induce IFN responses, particularly within microglia of the infected brainstem (56, 57). These innate immune responses serve as a critical first line of defense during the early stages of infection. However, in the context of neurodegeneration, excessive NLRP3 activation within the microglia of the 5xFAD (AD model) mouse brain accelerates disease progression (55, 58). While such responses contribute to neuropathology, several studies suggest that a balanced inflammatory response is necessary to protect neuronal progenitor cells from loss and the development of HSE (59, 60). However, it should be noted that much of this mechanistic evidence is derived from preclinical experimental systems, and direct validation in human brain tissue remains limited.

VZV also differentially affects glial biology depending on cell type and CNS region. Bubak et al. reported that VZV infection altered astrocyte morphology in a region-specific manner, leading to swollen, globular morphologies in hippocampal astrocytes but not in spinal cord astrocytes (61). Interestingly, VZV-infected spinal astrocytes showed attenuated proinflammatory responses, suggesting potential immune evasion strategies employed by the virus.

Similarly, SARS-CoV-2 infection induces microglial activation, promoting a shift toward DAM-like and IFN-responsive phenotypes. Moreover, SARS-CoV-2 exposure increases microglial synaptic pruning, which may contribute to neurological manifestations such as cognitive dysfunction and brain fog observed in post-COVID-19 syndrome (62). SARS-CoV-2-infected microglia also exhibit a proinflammatory phenotype (63). Intracerebroventricular infusion of the SARS-CoV-2 spike protein in mice induces delayed, long-term neuroinflammation in the hippocampus, specifically within the dentate gyrus, primarily via Toll-like receptor 4 (TLR4) signaling pathways, highlighting virus-specific mechanisms of glial activation and cerebral inflammation (64). Additionally, Kong et al. demonstrated that astrocytes were identified as a major cellular target in brain organoid and *in vitro* astrocyte models via neuropilin-1 (NRP1), leading to broad immune activation, including inflammatory responses, in brain organoid models and astrocytes (65). Importantly, astrocyte-mediated changes in the microenvironment resulted in bystander neuronal cell death, suggesting a potentially important crosstalk between glial cells and neurons. Similar to these clinical implications, SARS-CoV-2 infection alters the expression of AD-associated risk factor genes, including *IFITM3* and complement components, in both humans and mice (65).

Mechanistically, HIV-associated neurocognitive impairment is increasingly understood to arise from chronic microglia-driven neuroinflammation and metabolic dysregulation rather than direct neuronal infection. Supporting this, recent studies using a three-dimensional human neural organoid model incorporating microglia demonstrated that HIV-1 infection induces early immunometabolic and transcriptional reprogramming within the CNS, including activation of proinflammatory pathways, upregulation of chemokine signaling such as CCR6, altered amino acid metabolism, and enhanced mitochondrial metabolite transport (66). These findings suggest that HIV-infected microglia may establish a persistent inflammatory and metabolically dysfunctional CNS microenvironment that contributes to neuronal impairment and progressive cognitive decline (66). Importantly, modeling HIV neuropathogenesis has historically been limited by the lack of physiologically relevant *in vivo* systems, as conventional animal models often rely on artificial intracerebral viral inoculation and incompletely recapitulate human CNS infection. In this regard, a recently developed dual-humanized mouse model, generated by engrafting human induced pluripotent stem cells (iPSC)-derived microglia into the mouse brain together with human peripheral immune cells, represents a significant advance (67). By more faithfully mimicking the human route of infection, in which infected peripheral immune cells cross the BBB and establish CNS reservoirs, this platform offers an improved framework for dissecting chronic HIV-associated neuroinflammation, tracking latent viral reservoirs at single-cell resolution, and evaluating therapeutic strategies targeting persistent HIV-associated neurocognitive disorders (67).

### 3.2 Amyloidosis and tauopathy

**Figure 2:**
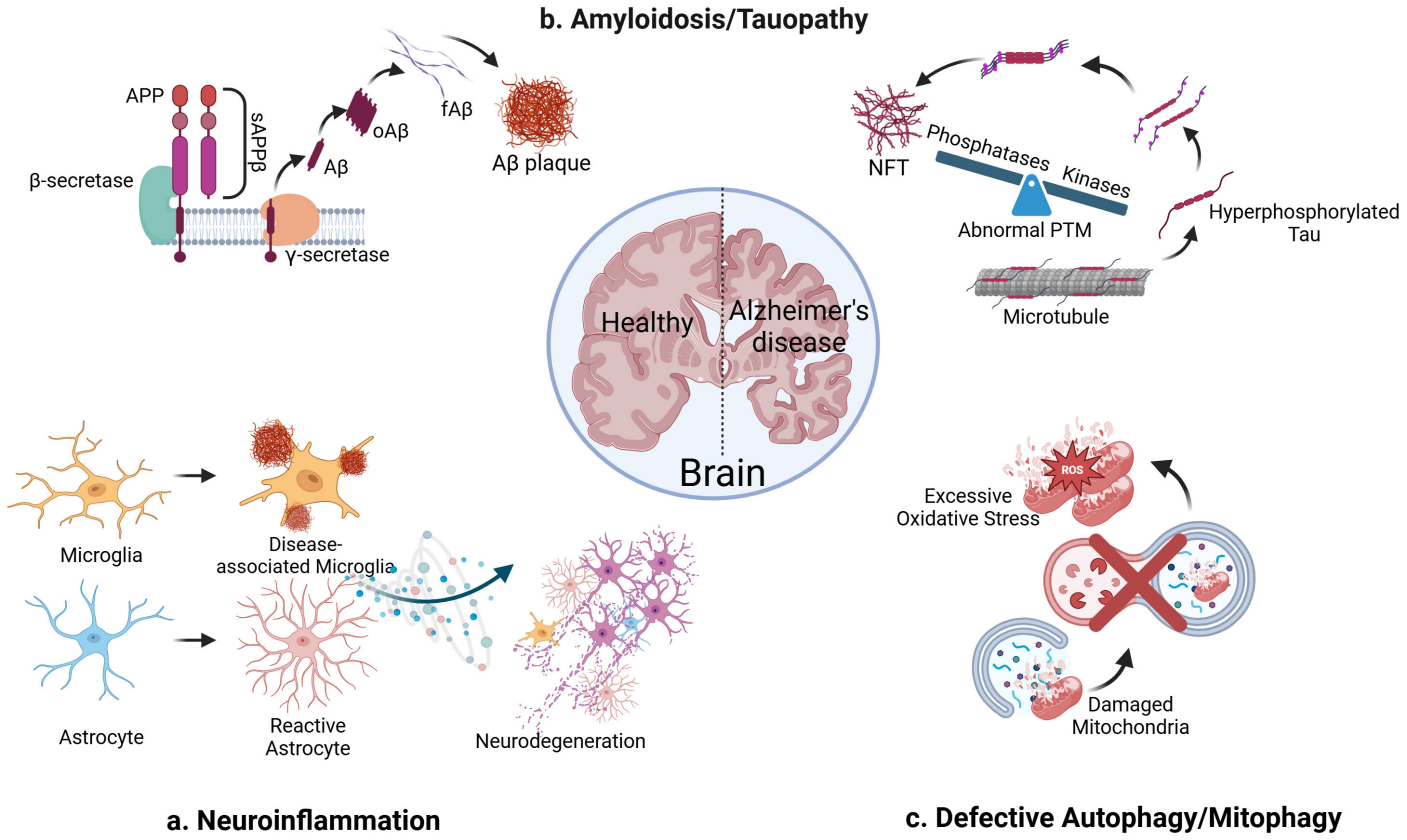
Possible mechanisms of viral triggers in neurodegeneration. **(a)** In neuroinflammation, disease-associated microglia and reactive astrocytes produce proinflammatory cytokines, leading to neurodegeneration. **(b)** Aβ, produced by sequential cleavages by β-secretase and γ-secretase of APP, oligomerizes as Aβ oligomers (oAβ), fibrillar form (fAβ), and Aβ plaque in amyloidosis. Hyperphosphorylated tau protein aggregates as neurofibrillary tangles (NFT) in tauopathy. **(c)** Defective autophagy/mitophagy function results in reduced phagocytosis of Aβ and accumulation of reactive oxygen species (ROS) generating mitochondria, thereby causing excessive oxidative stress.

Amyloidosis refers to a specific pathological condition triggered by the accumulation of Aβ, a hallmark phenotype of AD (**Figure 2**). Aβ is produced by the amyloidogenic pathway of the amyloid precursor protein (APP). Importantly, Aβ aggregates, including fibrils, are formed through the amyloidogenic pathway, which involves the sequential cleavages of APP by β-secretase (β-site APP-cleaving enzyme, BACE) and γ-secretase (68). The γ-secretase complex contains a catalytic subunit, presenilin 1 (PSEN1), together with nicastrin, Aph-1, and Pen-2 (69). Therefore, amyloidogenic pathway-related genetic mutations such as *APP*, *PSEN1*, and *PSEN2* (an isoform of PSEN1) serve as key indicators of insoluble Aβ formation. Given that neuronal lineage cells are known to actively process Aβ, many studies have examined the regulation of amyloidogenic processing and Aβ deposition in neuronal cells infected by neurotropic viruses.

Tau protein plays a major role in maintaining microtubule stability in neurons (70). However, tau exists in six different isoforms in the human brain and contains approximately 85 known phosphorylation sites. Tau phosphorylation is regulated by various kinases including GSK3 and CDK5, and phosphatases such as PP2A and PP1 (71). While phosphorylation of tau is necessary for its detachment from microtubules, hyperphosphorylated tau aggregates into neurofibrillary tangles (NFTs), which are a hallmark neuropathological feature of AD (72).

In the case of HSV-1 infection, several studies using brain organoids and *in vitro* systems have shown that HSV-1 alters the expression of genes involved in the amyloidogenic pathway (73). Consistently, HSV-1 infection induces upregulation of *PSEN1* and *PSEN2*, while downregulating *BACE* expression (74). Supporting these findings, Cairns et al. demonstrated in a 3D human brain-like tissue model that HSV-1 infection disrupts the amyloidogenic pathway, resulting in Aβ accumulation, hyperphosphorylated tau, and severe neuroinflammation (3, 75). A more recent study using the same model showed that even latent HSV-1 infection can lead to Aβ deposition and gliosis, particularly in the context of *APOE4* expression (3). In addition, although total *APP* levels may decrease during HSV-1 infection, 55-kDa C-terminal fragment of APP was significantly increased in HSV-1-infected neuroblastoma cells (76). Alongside Aβ aggregation, HSV-1 is also known to induce robust tau phosphorylation in brain organoids and primary cortical neurons by activating GSK3 (77–79). Interestingly, HSV-1-encoded ICP27 protein was found to co-localize with hyperphosphorylated tau at Ser396 (80). Moreover, CDK- and SRC/Fyn-mediated phosphorylated tau (p-tau) has been shown to exert antiviral effects against HSV-1 by reducing viral protein expression and preventing HSV-1-induced neuronal cell death. The involvement of NF-κB and IRF3 signaling pathways is suspected in this antiviral mechanism. Supporting this, D’Aiuto et al. reported nuclear accumulation of p-tau in neurons and neural precursor cells in HSV-1-infected cortical brain organoids (77). In addition, recent reports suggest the antimicrobial role of Aβ and p-tau during HSV-1 infection (81–83). Antimicrobial peptides (AMPs) restrict infections by bacteria, fungi, and viruses by entrapping microbes, and AMPs and Aβ share similar characteristics (84, 85). The infection of HSV-1 in the brains of AD transgenic mice resulted in the formation of plaques and NFTs, suggesting the antimicrobial role of Aβ and p-tau against viral infection in the brain (81, 82).

Previously, the direct link between VZV infection and Aβ production was not known (86). Bubak et al. showed that VZV-infected quiescent primary human spinal astrocytes exhibit the co-localization of VZV open reading frame (ORF) 63 and Aβ, which is thioflavin-T positive, detecting β-sheet structures from Aβ (86, 87). The supernatant of VZV-infected cells could also induce the aggregation of Aβ42 peptide into more elongated amyloid fibrils compared to the formation of condensed amyloid fibrils by the mock-infected control (86). Plasma samples from 14 human subjects with acute zoster infection were analyzed, and thioflavin-T positive amyloid was significantly increased in the zoster group, while there were no differences in the concentrations of Aβ42 and Aβ40 (87). In addition, measurement of cerebrospinal fluid (CSF) biomarkers showed that significantly decreased levels in Aβ42, Aβ40, and Aβ_42_:Aβ_40_ ratio and increased levels in total tau (t-tau) and p-tau at Thr181 in a small number of human patients, combined from 9 patients with HSV infection and 8 patients with VZV infection (88). When patient groups were separated, CSF p-tau increased significantly in the VZV group while CSF t-tau levels increased in the HSV group (88). CSF measurement in 128 adult patients with HIV showed that levels of t-tau and p-tau increased in the patient group with HSV-1 (37.5% of seroprevalence), but not in the group of HSV-2 (32%) nor VZV (45.3%) (89).

Recent studies suggest that patients with COVID-19 exhibited a reduced Aβ_42_:Aβ_40_ ratio and elevated levels of p-tau at Thr181 in plasma, indicating increased cerebral Aβ accumulation (43). Mechanistically, SARS-CoV-2 spike protein has been shown to promote Aβ accumulation in both *in vitro* neuronal cultures and retinal organoids (90). Notably, S2 domain of the spike protein interacts with the γ-secretase complex, leading to increased Aβ levels in hippocampal and cortical tissues (91). Furthermore, APP itself may facilitate SARS-CoV-2 entry into host cells, suggesting a vicious cycle in which Aβ pathology is both a cause and consequence of SARS-CoV-2 infection (92). Similar to these experimental findings, SARS-CoV-2 infection alters the expression of AD-associated risk factor genes, including *IFITM3* and complement components, in both humans and mice (65).

HIV infection has been shown to trigger Aβ production by altering the gene expression profile of the amyloidogenic pathway (93). Both *in vitro* and *in vivo* studies have demonstrated that the HIV-1-encoded transactivator of transcription (Tat) protein, an essential factor in HIV-induced neurotoxicity, interacts directly with APP (94). Importantly, HIV-1 downregulates the activity of neprilysin, a key Aβ-degrading enzyme, thereby promoting the accumulation of Aβ (95, 96). In a mechanism similar to that observed in SARS-CoV-2 infection, APP also binds to the HIV-1-encoded Gag protein, initially inhibiting viral particle production and spread. However, Gag protein counteracts this antiviral effect by promoting the processing of APP into neurotoxic Aβ, thus evading the host’s innate immune defense and contributing to neurodegeneration (97). Likewise, HIV-1 has also been implicated in tauopathy, particularly in aged individuals, as evidenced by elevated CSF tau/Aβ42 ratios, a key biomarker for AD diagnosis (98). HIV-1 induces tau phosphorylation through activation of GSK3, CDK5, JNK, and p38, notably increasing phosphorylation at Ser396 and Ser199 in a CCR5-dependent manner (99, 100). Furthermore, HIV-1 gp120 has been shown to promote tau pathology via activation of cGMP-dependent kinase II (101).

### 3.3 Autophagy/mitophagy dysregulation

Autophagy is closely linked to the degradation of Aβ and tau proteins associated with neurodegeneration, and dysregulation of autophagy is known to cause protein aggregation. In particular, autophagy and phagocytosis in microglia and astrocytes play essential roles in clearing Aβ, thereby reducing its accumulation (102–104). Autophagy-deficient microglia exhibit impaired capacity to engulf and degrade Aβ (102). Multiple viruses have evolved unique mechanisms to evade these host defense responses by subverting the autophagy or mitophagy process and further interfering with host antiviral signaling triggered by viral infection. Mitophagy is a selective, mitochondria-specific autophagy process that sequesters damaged or dysfunctional mitochondria into autophagosomes, which are double-membrane vesicles that ultimately fuse with lysosomes for degradation of the sequestered cargo (105). Overall, mitophagy is triggered upon mitochondrial membrane potential depolarization and regulated by a ubiquitin-dependent, PINK1/Parkin pathway, or ubiquitin-independent, receptor-mediated pathway (106, 107).

HSV-1 has been well studied for its contribution to neurodegenerative phenotypes via impairment of mitophagy in microglia (54, 108). Consistent with this, HSV-1-mediated inhibition of mitophagy leads to mitochondrial dysfunction and damage, suppressing phagocytosis of Aβ and other substrates by downregulating key phagocytic receptors, including triggering receptor expressed on myeloid cells 2 (TREM2) (54). Notably, HSV-1 appears to specifically target microglial TREM2 to inhibit phagocytosis of neurons and antiviral responses through the TREM2-STING signaling axis (109). HSV-1 encodes several proteins that inhibit autophagic processes. For instance, US11, which is a kinase expressed at late times after infection, inhibit PKR-inducible and TBK1-mediated autophagy through targeting PKR and TRIM23 (110, 111). ICP34.5 interacts with Beclin-1 to inhibit autophagosome formation (108, 112). US3 also downregulated autophagy and mitophagy by inducing phosphorylation of ULK1 at inhibitory site, Ser757 and interacts with Beclin-1 (113). Furthermore, mitophagy-inducing agents with antiviral properties may serve a dual-purpose pharmacologic intervention and may suppress latent viral reservoirs and prevent reactivation events that exacerbate neurodegenerative pathology (54). Interestingly, recent studies show that VZV similarly modulates autophagy in neuronal cells, yet with an opposite effect on viral fitness (114). These observations suggest that autophagy or mitophagy may represent a potential mechanistic link between viral infection and neurodegeneration. Similarly, recent studies have shown that SARS-CoV-2 and HIV-1 infections have been shown to modulate PINK1/Parkin-mediated mitophagy, which dampens microglia-mediated neuroinflammation (115–118).

Recent studies have identified a novel astrocyte-neuron pathway of HIV-associated neurotoxicity, in which co-exposure to the HIV-1 Tat protein and cocaine induces a hypermetabolic state and excessive mitochondrial fission in astrocytes, promoting the release of extracellular vesicles containing fragmented mitochondria (119). These dysfunctional mitochondria can subsequently be transferred to neighboring neurons, where they impair neuronal bioenergetics and suppress electrophysiological activity, suggesting a previously underappreciated mechanism of non-cell-autonomous neuronal injury in HAND, particularly in the context of substance abuse (119). Complementing this, mechanistic studies in microglia have demonstrated that HIV-1 Tat directly induces mitochondrial damage and initiates mitophagy, but ultimately disrupts mitophagosome maturation and autophagic flux, resulting in the accumulation of dysfunctional mitochondria and exaggerated proinflammatory cytokine release (118). Together, these findings suggest that HIV-associated neuropathogenesis may involve both aberrant mitochondrial dynamics and defective mitochondrial quality control across distinct glial populations (118, 119). However, while these studies support a role for mitochondrial dysfunction in HAND, further investigation is needed to determine how broadly canonical mitophagy impairment contributes to chronic neurodegeneration *in vivo*, particularly in physiologically relevant humanized models (67).

## 4 Conclusion

The “Infection hypothesis” of AD has recently emerged as a major focus of scientific attention. Growing evidence suggests that viral infections may contribute to neuroinflammatory processes associated with neurodegeneration. Neurotropic viruses such as herpesviruses, SARS-CoV-2, and HIV-1 are capable of establishing chronic infection/neuroinflammation in the CNS, and are frequently associated with subsequent cognitive impairment and neurodegeneration. In particular, HSV-1 is characterized by its ability to enter a latent state and reactivate during aging or periods of systemic stress. Therefore, preventing initial viral infection in the peripheral nervous system and/or the CNS, as well as mitigating the course of viral infection and managing neuroinflammation, may represent potentially important strategies. It is still not well known what exact mechanisms of viral infection could trigger a cascade of events into chronic viral infection and eventually neurodegeneration. Potential therapeutic strategies may include not only epidemiological ones, including 1) limiting viral infection to block viral entry to host cells, and 2) vaccination as a prevention approach, but also targeting cellular mechanisms, including 1) reducing Aβ and phosphorylated tau productions, 2) mitigating acute and chronic neuroinflammation, and 3) targeting autophagy/mitophagy for better functions. In addition, understanding the mechanisms of latent viral infection and reactivation is crucial for elucidating their potential impact on neurodegenerative disorders in the future. Because herpesviruses remain dormant within the host for decades, it is imperative that future research investigates neurodegenerative phenotypes specifically within viral reactivation models rather than solely in acute infection contexts. These growing research fields in the viral-immune axis in the brain can further enhance the understanding of the impact of chronic infection and inflammation in the CNS and provide more systematic, innovative, and targeted therapeutic approaches to combat neurodegeneration.
